# Comment on: “Effects of Monocular Flicker on Binocular Imbalance in Amblyopic and Nonamblyopic Adults”

**DOI:** 10.1167/iovs.66.2.29

**Published:** 2025-02-11

**Authors:** Alexandre Reynaud

**Affiliations:** 1 McGill University, Research Institute of the McGill University Health Centre, Montréal, Canada. E-mail: alexandre.reynaud@mcgill.ca

In their recent article “Effects of Monocular Flicker on Binocular Imbalance in Amblyopic and Nonamblyopic Adults,” Lu and colleagues^1^ provide very interesting results on binocular combination when one eye's view is obstructed at various temporal frequencies. They show that monocular flicker in the range of 4 to 20 Hz diminishes the contribution of the flickered eye, with larger effect at low temporal frequency (the lower the frequency, the weaker the contribution).

They discuss their results and suggest that they could be explained by a “patching effect,”^2^^,^^3^ resulting from the temporal flickering, that would modulate the contrast gain of the obstructed eye. While this explanation sounds perfectly plausible in light of recent studies on that matter,^4^^,^^5^ I would like to suggest another one, simply based on the temporal integration required in the visual system; a burning topic in binocular vision. I explicate a simplified model of temporal integration that can faithfully account for their results.^6^

Across all the neurons and connections that constitute it, the visual system is progressively integrating the visual information over time, with dynamics characterized by an impulse response function, IRF (i.e. the temporal integration window). In the visual system, this IRF is commonly modeled as a gamma probability density function (Equation 1) with a scale parameter (roughly corresponding to the width of the integration window) in the range of 50–100 ms (Fig., top-middle^7^^–^^11^):

**Figure.**
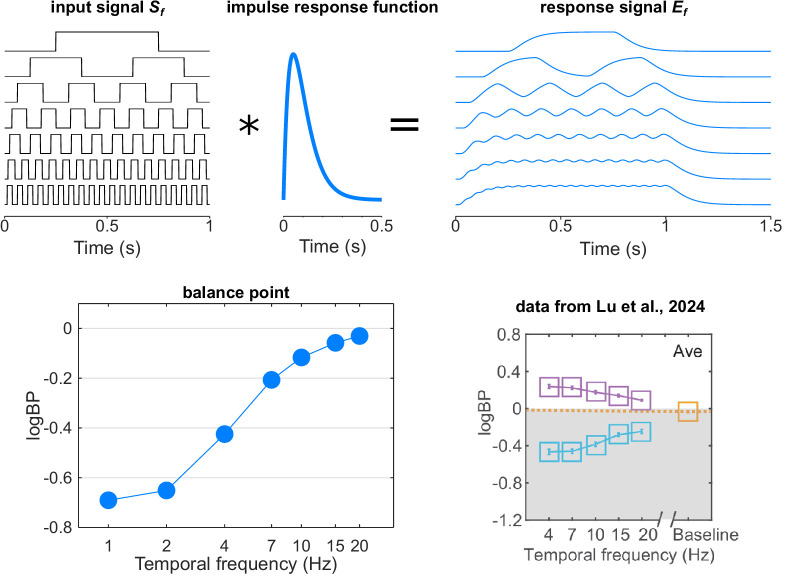
**Top row:** convolution of the squarewave input signals **(left)** by the impulse response function of the system **(middle)**, resulting in smoothed signals **(right)**. Note that the amplitude of the signals is normalized. **Bottom row: Left:** calculated balance points at different temporal frequencies. **Right:** Measured balance points from Lu et al.^1^ The *blue* and *purple squares* represent the logBP measured with flicker applied to the nondominant eye and dominant eye, respectively. The *yellow square* represents the baseline level with no flicker. Panel reproduced from Lu et al.^1^

The following simulations were performed using Matlab 2023a (The MathWorks), using the gamma probability density function gampdf with a shape parameter *k* = 2 and scale *θ* = 50 ms. The code is available at https://mvr.mcgill.ca/AlexR/data_en.html.

(1) $$IRF\left(t\right)=gampd{f_{k,}}_{\theta }\left(t\right)$$

The visual input flickering (Fig., top left) would then be temporally integrated by the visual system, through its IRF, resulting in a smoothed response signal *E_f_* (Fig., top right, convolution between the squarewave input *S_f_* and the IRF of the system):

(2) $$E_{f}\left(t\right)=S_{f}\left(t\right)*IRF\left(t\right)$$

With *S_f_* the squarewave input at temporal frequency *f* and IRF the impulse response function of the system as defined in Equation 1. This filtering stage will modulate the shape of the envelope of the integrated response as a function of the temporal frequency of the input signal. The total response of one eye *R_f_* at a given temporal frequency *f* would then be characterized by the cumulative sum of the normalized signal over time:

(3) $$R_{f}=\int_{t}\;{\bar{E}}_{f}\left(t\right)$$

The ratio between the response of that eye's signal and the constant input received by the other eye, would characterize the balance point, expressed in logarithmic units as in Lu et al.^1^:

(4) $$logBP=-log\left(1/R_{f}\right)$$

The simulated balance points as a function of the temporal frequency in the range of 1 to 20 Hz are presented in the bottom-left panel of the Figure. The balance point monotonously increases with temporal frequency, almost reaching 0 at high temporal frequency. This fairly resembles the observations of Lu et al.,^1^ who observed a balance point increasing from −0.4 dB at 4 Hz to −0.2 dB at 20 Hz in the control population (blue squares in Fig., bottom right). The remarkable consistency of their observations in both the control and amblyopic populations also suggests the implication of low-level mechanisms.

When integrating the visual information over time, over multiple processing stages, our visual system is somehow filtering the visual signal. For instance, this is one of the reasons why we don't perceive the flicker on television monitors. This temporal integration takes time. In primates, the visual information reaches the primary visual cortex in approximately 50 ms, and the higher visual areas only after 200 ms.^12^ In this communication, I have presented how this integration will modulate the final envelope of the integrated response as a function of the temporal frequency of the input signal. In the eye presented with a high temporal frequency flicker, the signal will be smoothed out, with very little loss. So the integrated signal will present a shape almost as if the input was continuous, as in the other eye.
